# Health-Related Quality of Life, Fatigue, Level of Physical Activity, and Physical Capacity Before and After an Outpatient Rehabilitation Program for Women Within Working Age Treated for Breast Cancer

**DOI:** 10.1007/s13187-022-02211-6

**Published:** 2022-08-16

**Authors:** Gunhild M. Gjerset, Tone Skaali, Mette Seland, Lene Thorsen

**Affiliations:** 1 Unit for Psychosocial Oncology, Coping and Rehabilitation, Department of Clinical Service, Division of Cancer Medicine, Oslo University Hospital, P.O. Box 4953 Nydalen, 0424 Oslo, Norway; 2 National Advisory Unit On Late Effects After Cancer Treatment, Department of Oncology, Division of Cancer Medicine, Oslo University Hospital, Oslo, Norway; 3 Department of Clinical Service, Division of Cancer Medicine, Oslo University Hospital, Oslo, Norway

**Keywords:** Breast cancer, Outpatient rehabilitation program, Patient education, Physical activity

## Abstract

The aims were to examine changes in patient-reported outcome measures (PROMs), level of physical activity (LPA), and physical capacity from before to after an outpatient rehabilitation program (ORP) for women with breast cancer (BC). Further aims were to explore the proportions of patients with clinically relevant improvements defined as ≥ 10% beneficial change in the scores of PROMs and variables associated with such improvements.

A total of 270 women within working age (< 67 years) who recently (< 1.5 years) had completed primary therapy for BC with curative intent were included. The ORP consisted of seven weekly group sessions with patient education, group conversations, and PA. The patients completed questionnaires measuring health-related quality of life (HRQoL), fatigue and LPA before (T0), immediately after (T1), and 6 months after (T2) the program, and were physically tested at T0 and T1. The mean age of the patients was 50.4 years (SD 7.3) and the mean time since diagnosis was 10.6 months (SD 2.6). All patients had undergone surgery and 94% radiotherapy, and 96% had received chemotherapy and/or hormonal therapy.

Physical-, role-, emotional-, cognitive-, and social function, global health, and fatigue significantly improved from T0 to T1. Physical-, role-, and cognitive function, and fatigue significantly improved from T1 to T2. LPA and physical capacity significantly improved from T0 to T1. More than 40% of the patients had a clinically relevant improvement in role-, social function, and fatigue symptoms, from T0 to T1. Low level of education was associated with an improvement in emotional function, and living alone was associated with an improvement in mental fatigue.

HRQoL, fatigue, LPA, and physical capacity improved in women within working age recently treated for BC who participated in an ORP.

**Supplementary Information:**

The online version contains supplementary material available at 10.1007/s13187-022-02211-6.

## Introduction

Breast cancer (BC) patients are at risk of several physical and/or psychosocial adverse effects after anti-cancer treatment (Runowicz et al. 2016). Some of the most common adverse effects after BC treatment are fatigue, impaired arm function, neuropathy, menopausal symptoms, lymphedema, musculoskeletal symptoms, pain, weight gain, and emotional distress (Runowicz et al. 2016; Moore 2020). Facing these health issues might have a large negative influence on physical- and mental well-being, resulting in reduced health-related quality of life (HRQoL) and reduced work-capacity (Park et al. 2019; Hauglann et al. 2012).

To prevent or mitigate the adverse effects, several in- and outpatient rehabilitation programs have been developed. The type and extent of rehabilitation needed depend on the complexity of the adverse effects. Components often suggested in rehabilitation programs for BC patients are physical activity and exercise, yoga, lymphedema treatment, and psychosocial interventions (Olsson Möller et al. 2019). Meta-analyses indicate that multidisciplinary interventions, consisting of both physical and psychosocial components, have positive effects on fatigue and physical function in BC survivors (Myrhaug et al. 2018; Scott 2013). A recent systematic review also indicated that multidisciplinary outpatient programs in different cancer types improve physical and psychosocial outcomes (Kudre et al. 2020). Moreover, studies have demonstrated positive effects of exercise programs in BC populations after treatment on fatigue, muscle strength, cardiorespiratory fitness, physical function, and HRQoL (Campbell et al. 2019; Lahart et al. 2018).

BC is the most frequent cancer in women, and a large proportion of these women are within working age (18 − 67 years in Norway) when diagnosed (Cancer Registry of Norway 2019). It is important to provide BC patients within working age less time-consuming programs aiming to regain their physical and psychosocial function, so they can be able to resume to or continue with everyday life with work, studies, childcare, and family without having to be away from home for several weeks. Such outpatient rehabilitation programs (ORP) are also less expensive for the society compared to inpatient rehabilitation. Studies investigating the benefits of cost- and time- effective multidisciplinary ORP in BC patients tailored to women within working age are limited.

The aims of the present study were to examine changes in HRQoL, fatigue, and level of physical activity from before to after an ORP in women with BC within working age after primary treatment with curative intent. Further aims were to study changes in physical capacity before and immediately after the program and the proportions of patients with a clinically relevant improvement in subscales of HRQoL and fatigue, and to explore demographic-, medical-, and health characteristics of those with such improvements. We hypothesized that the scores of HRQoL, fatigue, level of physical activity, and physical capacity would improve during the ORP.

## Material and Methods

### Design and Patients

This is a pre-post intervention study, conducted at the Cancer Rehabilitation Unit at Oslo University Hospital (OUH) in Norway between January 2012 and May 2016. Women with BC, within working age (18–67 years), on or in risk of being on sick-leave and who had been referred to the ORP, were invited. Those who were diagnosed > 24 months ago, those who were currently receiving cancer treatment (except for hormonal therapy and traztuzumab), and those who had received treatment without curative intention were excluded from the present analyses.

### The Outpatient Rehabilitation Program (ORP)

**Fig. 1:**
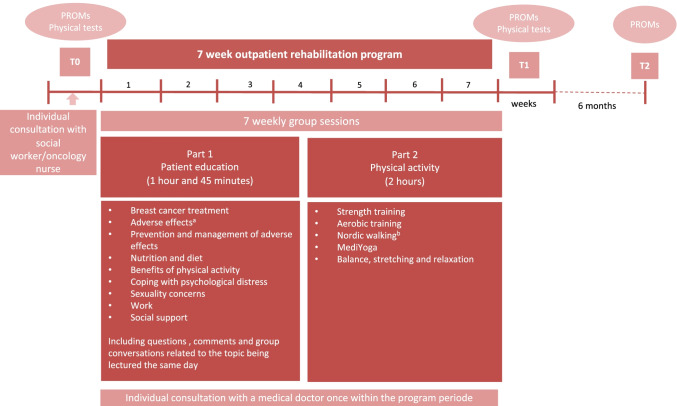
The outpatient rehabilitation program. PROMs, patient-reported outcome measures. ^a^Such as lymphedema, fatigue, and neurotoxicity; ^b^Outdoor walking with poles

The ORP is presented in Fig. 1. The overall goal of the ORP was to teach the patients to manage and cope with their perceived cancer-specific adverse effects. The ORP included two individual consultations and seven group sessions. The individual consultations included a 1-h consultation in the beginning with a social worker or an oncology nurse to assess challenges and goals for the rehabilitation period, and each patient also received a 1-h consultation later in the ORP with a medical doctor focusing on the rehabilitation process. The group sessions included seven once-a-week meetings with duration of 4–5 h with a group of 9 to 15 patients. Each day consisted of the components: a patient education session, group conversation during the lecture, and a physical activity session. The patient education sessions included relevant topics, and were led by specialists within the different themes: oncologist, dietician, psychologist, physiotherapist, social worker, and nurse/sexologist. During and at the end of each education session, the patients could ask questions to the specialist, and comment on and share their experiences related to the topics in the lecture. This was organized as a steered group conversation. After the lecture with the group conversation, the patients participated in different types of physical activities. The overall aim was to introduce different physical activities to the patients hoping that they would find a preferable activity to continue with after the end of the ORP.

### Assessments

The patients completed questionnaires before (T0), immediately after (T1), and 6 months after (T2) the ORP. Physical tests were performed at T0 and T1.

### Patient-Reported Outcome Measures (PROMs)

HRQoL was assessed by the European Organization for Research and Treatment of Cancer Quality of Life Questionnaire (EORTC QLQ-C30) (Aaronson et al. 1993). The EORTC QLQ-C30 contains five functional scales (physical-, role-, emotional-, cognitive-, and social function), symptom scales (fatigue-, pain-, and nausea), and a global health/QoL scale. All scales were calculated according to the EORTC QLQ-C30 manual and transferred into a 0–100 scale (Fayers 2001). Higher scores represent better function and more symptoms. A clinically relevant improvement was defined as a beneficial change corresponding to ≥ 10% of the maximum scale score in each scale of EORTC QLQ-C30 (i.e., ≥ 10 points in each sub scale) (Osoba et al. 2005).

Fatigue was assessed by the Fatigue Questionnaire (FQ) (Chalder et al. 1993). FQ consists of seven items covering physical fatigue and four items covering mental fatigue. Each item has four response alternatives scored from 0 to 3, with higher scores indicating higher levels of fatigue. Summarized scores for physical fatigue range from 0 to 21, mental fatigue from 0 to 12, and total fatigue (sum of physical and mental fatigue) from 0 to 33. A beneficial change corresponding to ≥ 10% of the maximum scale score in each scale (i.e., ≥ 2.1 point-change in physical fatigue, ≥ 1.2 point in mental fatigue, and ≥ 3.3 point in total fatigue) was defined as a clinically relevant improvement (Nordin et al. 2016).

Level of physical activity was assessed by the Nord-Trøndelag Health Study Physical Activity Questionnaire (HUNT-1 PA-Q) (Kurtze et al. 2008). HUNT-1 PA-Q consists of three questions regarding frequency, duration, and intensity of their physical activity defined as walking, swimming, skiing, exercising, or participating in organized sports. The patients were asked the following: “How often do you exercise during a typical week?” (“never,” “less than once a week,” “2–3 times per week,” “nearly every day”), “For how long do you exercise (average min per session)?” (“less than 15 min,” “15–29 min, 30 min–1 h,” “more than 1 h”), and “How hard do you exercise (on average)?” (“no sweating or without losing breath,” “losing breath and sweating,” “to near exhaustion”). The three components were calculated into a physical activity summary index ranged from 0 (no activity) to 15 (maximum activity) according to Kurtze et al. (Kurtze et al. 2008).

### Physical Tests

Physical capacity was assessed by the incremental shuttle walk-test (ISWT) (Singh et al. 1992). The patients are instructed to walk back and forth a 10-m distance. The walking speed begins with 1.8 km/h and is increased by a 0.6 km/h increment every minute. The speed is externally paced and controlled by a beep-signal played from a CD-player (Singh et al. 1992). There are 12 levels in total and each level lasts for 1 min. The test is stopped when the participant feel too breathless or fatigued to continue the required speed to complete a 10-m shuttle interval within the time frame. After the test, the total distance walked is summed (range 0–1020 m). Muscle strength was measured by one repetition maximum (1RM) in leg press and chest press.

### Background Variables

Background variables were self-reported or collected from the medical database. Demographic variables included age, civil status (living as a couple/living alone), education (high > 13 years/low ≤ 13 years), and working full-/part time (yes/no). Medical variables included type of treatment (local treatment [surgery ± radiotherapy]/local and systemic treatment [chemotherapy and/or hormone therapy + surgery ± radiotherapy ± others]), time since diagnosis (months), time since radiotherapy (months) (represents end of the most intensive treatment period), and comorbidity defined as any long lasting (> 12 months) physical and/or mental condition which had led to reduced daily function before the cancer diagnosis (yes/no). Health variables included current smoking daily or occasionally (yes/no) and weight and height to calculate BMI: overweight/obese (BMI ≥ 25) (kg/m^2^) (yes/no).

### Statistical Analysis

Mean changes in HRQoL, fatigue, and level of physical activity from T0 to T1 and from T1 to T2 were analyzed with paired sample *t* test. The proportions of patients with clinically relevant improvements (i.e., ≥ 10% improvement of the maximum scale score from T0 to T1) were calculated only for the outcomes that improved statistically significant from T0 to T1. Logistic regression analyses were used to evaluate demographic-, medical-, and health factors significantly associated with a clinically relevant improvement (versus no clinically relevant improvement). Baseline scale scores and variables statistically associated with the outcome variables in the univariate analyses were included as explanatory variables in multivariate regression analyses. A *p* value less than 0.05 was considered statistically significant. Adjusted odds ratios (aOR) were presented with 95% confidence intervals (95% CI). The analyses were performed using SPSS version 25.0 for Windows (SPSS Chicago, IL).

### Ethics

The study was a quality improvement study conducted at OUH (ePhorte number 2013/7933), and considered outside the mandate of the South-East Regional Committee for Medical and Health Research Ethics. According to the Personal Data Act, the legal basis for processing personal and health information in the project is the General Data Protection Regulation article 6 number 1 (a) and article 9 number 2 (j). The Privacy and Data Protection Officer at OUH evaluated the study and recommended the personal data and health information processing. Written informed consent was provided from all participating patients.

## Results

Of the 294 who completed the questionnaire at T0, 270 completed the questionnaire both at T0 and T1, and 242 completed the questionnaire at all time points T0, T1, and T2.

### Baseline Characteristics

Baseline characteristics of the sample are presented in Table 1. Mean age of the patients was 50.4 (SD 7.3) (range 30–65), 75% had a high education, 30% worked full or part time, 96% had undergone systemic treatment, and the mean number of months since diagnosis was 10.6 (SD 2.6) (range 4.1–24.2).

**Table 1:** Baseline characteristics of the participants (*n* = 270)

Variables	*n* (%)
Demographical characteristics	
Age (years) Mean (SD) (range)	50.4 (7.3) (30–65)
Civil status Living as a couple Living alone	202 (75) 68 (25)
Education (*n* = 268) High (> 13 years) Low (≤ 13 years)	201 (75) 67 (25)
Work full/part time No^a^ Yes^b^	188 (70) 82 (30)
Medical characteristics	
Treatment Local treatment^c^ Local and systemic treatment^d^	12 (4) 258 (96)
Months since diagnosis Mean (SD) (range)	10.6 (2.6) (4.1–24.2)
Months since radiotherapy (*n* = 255) Mean (SD) (range)	2.3 (1.6) (–0.9 to 9.2)
Comorbidity^e^ (*n* = 269) No Yes	211 (78) 58 (22)
Health characteristics	
Smoking (daily or occasionally) No Yes	249 (92) 21 (8)
Overweight/obese (BMI (kg/m^2^) ≥ 25) (*n* = 255) No Yes	150 (59) 105 (41)

Numbers may not add up to 270 because of missing data

^a^100% sick-leave/disability benefit/social support/unemployed

^b^Of those 82 who worked (but in risk to be on sick-leave), 6 (1%) worked full-time and 76 (99%) worked part-time

^c^Surgery ± radiotherapy

^d^Chemotherapy and/or hormonal therapy + surgery ± radiotherapy ± others

^e^Comorbidity defined as any long lasting (> 12 months) physical and/or mental condition which had led to reduced daily function before the cancer diagnosis

### PROMs

Changes in PROMs are presented in Table 2. The mean scores of physical-, role-, emotional-, cognitive-, and social function and global health/QoL status improved significantly during the ORP, and physical-, role-, and cognitive function continued to improve significantly 6 months after the ORP. Fatigue symptoms measured by EORTC QLQ C-30 and physical-, mental-, and total fatigue measured by FQ were significant lower at the end of the ORP than before. Six months after the ORP, these levels had continued to decrease. No changes were found for pain symptoms. Level of physical activity increased significantly during the ORP. From the end of the ORP to follow up 6 months after, the level of physical activity had significantly decreased.

**Table 2:** Changes in subjective measures from T0 to T1 and T1 to T2 and objective measures from T0 to T1 (*n* = 242)

			**T0**	**T1**	**T2**		**From T0 to T1**		**From T1 to T2**	
	***﻿n***		Mean (SD)	Mean (SD)			***﻿p***		***﻿p***	
Subjective measures										
HRQoL, EORTC QLQ-C30										
Physical function	241		78.0 (15.5)	81.3 (13.3)	83.4 (14.7)		< 0.001		0.009	
Role function	240		56.3 (28.8)	61.7 (27.4)	69.3 (28.2)		0.003		< 0.001	
Emotional function	240		72.0 (24.0)	74.7 (22.2)	76.1 (21.3)		0.028		0.310	
Cognitive function	240		64.0 (24.6)	67.1 (24.2)	71.0 (25.2)		0.019		0.003	
Social function	240		59.6 (27.5)	64.8 (25.3)	66.9 (27.8)		< 0.001		0.231	
Global health/QoL status	239		60.2 (18.9)	64.3 (19.8)	66.0 (22.2)		< 0.001		0.191	
Fatigue symptoms	241		49.3 (23.9)	45.3 (23.3)	41.9 (24.5)		0.003		0.015	
Pain	242		31.3 (27.4)	28.9 (25.9)	30.0 (28.6)		0.120		0.455	
Fatigue, FQ										
Total fatigue	240		20.9 (4.8)	19.2 (5.2)	17.9 (5.5)		< 0.001		< 0.001	
Physical fatigue	241		13.7 (3.5)	12.5 (3.7)	11.5 (3.9)		< 0.001		< 0.001	
Mental fatigue	240		7.2 (2.0)	6.7 (2.1)	6.3 (2.3)		< 0.001		0.020	
Physical activity level, HUNT-1 PA-Q										
Physical activity summary index	236		4.1 (2.7)	4.7 (2.7)	4.3 (2.7)		< 0.001		0.028	
Objective measures										
ISWT (meter), total length	236		790.8 (195.6)	861.3 (168.7)			< 0.001			
Maximal muscle strength (kg)										
Leg press	212		121.0 (32.6)	133.4 (33.8)			< 0.001			
Chest press	135		26.6 (10.0)	30.2 (10.8)			< 0.001			
Numbers may not add up to 242 because of missing data HRQoL: Health-related quality of life										
EORTC QLQ-C30: European Organization for Research and Treatment of Cancer Quality of Life Questionnaire. Increasing scores imply better function and more symptoms										
FQ: Fatigue Questionnaire. Increasing scores imply more fatigue										
HUNT-1 PA-Q: Nord-Trønderlag Health Study Physical activity Questionnaire. Increasing scores imply higher activity										
ISWT: The incremental shuttle walk-test										

### Physical Capacity

Total distance measured by ISWT increased significantly with 70.5 m during the ORP. Muscle strength measured by 1 RM improved significantly with 12.4 kg and 3.6 kg in leg press and chest press, respectively (Table 2).

### Clinically Relevant Improvements in PROMs and Associated Factors

The proportion of patients with clinically relevant improvements in the PROMs subscales from T0 to T1 ranged from 22% (mental fatigue) to 46% (role function). More than 40% of the patients had clinically relevant improvements in role-, social function, and fatigue symptoms measured by the EORTC QLQ-C30 (Online Resource 1).

A worse baseline score was associated with having a clinically relevant improvement in all PROMs subscales. In unadjusted analyses, patients with comorbidity and patients with an unhealthy BMI were more likely to experience clinically relevant improvement in their physical function after participating in the ORP (Online Resource 2). Those that live alone and those with a lower education experienced more often a clinically relevant improvement in emotional function after participating in the ORP (Online Resource 3). Patients with an unhealthy BMI were more likely to experience clinically relevant improvement in physical fatigue (Online Resource 4), and those that live alone were more likely to experience clinically relevant improvement in mental fatigue after participating in the ORP (Online Resource 5).

Multivariate analyses showed that patients with a lower education more often experienced clinically relevant improvement in their emotional function after participating in the ORP (Online Resource 3). In addition, patients that live alone were more likely to experience clinically relevant improvement in their mental fatigue after participating in the ORP (Online Resource 5).

## Discussion

The present study show that BC patients within working age participating in a time- and cost-effective ORP, including patient education sessions, group conversations, and physical activities, achieve statistical significant improvements in HRQoL, fatigue, physical activity level, and physical capacity. Clinically relevant improvements were found in PROMs subscales for more than 40% of the patients. Low level of education was associated with a clinically relevant improvement in emotional function, and living alone was associated with a clinically relevant improvement in mental fatigue.

### PROMs

Prospective studies have shown that HRQoL increases during the first 6 months to a year after BC surgery (De Gournay et al. 2013; Moro-Valdezate et al. 2013). A study using EORTC QLQ C-30 found that global health/QoL scale increased in average 5–7 points during the first year after BC surgery (De Gournay et al. 2013). Our group of BC patients increased, in a more time-effective way, in average 6 points in global health/QoL scale during the OPR. Baseline level of the global health/QoL scale was lower in our group compared to the non-intervention group that could implicate that we have reached those with an impaired global health/QoL who are in need of rehabilitation.

Our results showed that role function improved the most, and it continued to improve also 6 months after end of the ORP. A possible explanation for this positive trend could be that during the ORP, the patient received a lecture on work that might have affected the patients’ perspectives on the importance of work and also how to find a balance concerning work and rehabilitation. Furthermore, the fact that the BC patients in the present study were highly educated, in working age, and that most of them had a connection to work before their diagnosis might have contributed.

To compare our findings directly with other studies would be preferable in terms of outcome measure, type of patients, time since diagnosis, and type of program (content, duration, frequency, etc.). In line with our findings, a study conducted in the Netherland on mainly BC patients (84%) undergoing chemotherapy found that HRQoL and fatigue were improved after a 12-week outpatient multidisciplinary program with combined occupational counselling (one–three sessions in total) and supervised physical activity (twice weekly) (Leensen 2017). Leclerc and colleagues found significant improvements in all EORTC function and for most symptom scales after a 12-week outpatient rehabilitation program including physical activity (three sessions a week) and psycho-educational (one session a week) among women with BC after the end of treatment (≤ 1 year) compared to a control group (Leclerc et al. 2017). A municipality-based Danish outpatient rehabilitation program for women with BC, including physical activity twice weekly for 16 weeks and after individual needs services such as physiotherapy, occupational therapy, patient education, dietary counseling, social counseling, and smoking cessation, demonstrated increases in HRQoL (Rossen et al. 2019). Our results might indicate that 1 day for 7 weeks could be enough to achieve improvements.

We found that some subscales of function (physical-, role-, and cognitive function) and fatigue still continued to improve 6 months after the program. In comparison, Leclerc et al. (Leclerc et al. 2018) found that the improvements maintained and were significantly better than the control group at 3- and 6-month follow-up after the rehabilitation program.

The physical activity level increased significantly during the ORP period; however, from the end of the ORP to 6 months after the physical activity level, it decreased significantly. This emphasizes the importance of follow-up support by for example a phone call, a follow-up group meeting, or a plan for further exercise for the period after the ORP.

### Physical Capacity

Our results indicate that the patients accomplishing the ORP significantly improved physical capacity measured by ISWT in walking distance and muscle strength in leg press and chest press. In line with our findings, Leclerc and colleagues (Leclerc et al. 2017)found significantly improvements in walking distance measured by The six-minute walk test after their 12-week rehabilitation program. However, Leensen et al. (Leensen 2017) found no effect on VO_2_peak after the 12-week program, but that could be explained by the main focus for the exercise program was to improve muscle strength. In comparison to our findings, Leensen and colleagues also showed significantly improvements of muscle strength in leg press and deltoid pulley (Leensen 2017).

### Clinically Relevant Improvements in PROMs

In line with findings from a Danish study (Rossen et al. 2019), we observed that the patients with the worse HRQoL and fatigue at baseline had clinically relevant improvements in these outcomes. Furthermore, Rossen et al. found that 55% had a clinically relevant improvement in HRQoL measured by overall FACT-B score ≥ 8 points increase from entry to end of rehabilitation, whereas we found the percentage to be a little less, varying from 22 to 46% in the different PROMs subscales. This could be explained by the longer duration of the rehabilitation in the Danish study and more individual services offered based on their needs compared to our study. Rossen et al. also found that the group of patients with the lowest HRQoL had significantly longer courses of rehabilitation and participated in more activities totally. We found the highest proportion of patients with clinically relevant improvements in role function, social function, and fatigue symptoms (46%, 42%, and 44%, respectively). The potential explanations for the finding on role function are stated previously. An explanation to the benefits found in social function could be the type of setting of the ORP, where the social connection in the group, consisting of the same 9–15 participants over a period of time, is of importance including lunches, lectures, physical activity, and group conversations all together. The clinically relevant improvements in fatigue symptoms could be explained by the physical activity program accomplished. The effect of physical activity on fatigue level is well documented in the literature (Mustian et al. 2017).

We found few demographic, medical, and health variables associated to clinically relevant improvements. We had expected to see more factors related to the clinically relevant improvements, even though this was an explorative part of the study. Those with low level of education were almost three times more likely to obtain a clinically relevant improvement in emotional function compared to those with a high educational level. Also, those living alone were twice as likely to have a clinically relevant improvement in mental fatigue compared to those living as a couple. This could indicate that those with a lower socioeconomic status and living alone have more advantage of the program, maybe because of the social aspects of it and the potential they have to improve. Clinicians should assess the need for rehabilitation across all groups of cancer patients, and follow up with referral of patients who are assumed to benefit of such programs. The short outpatient rehabilitation program used in this study is not only cost- and time-effective for the society, but also for the patients. For those who need rehabilitation, it could be a dilemma to prioritize it concerning the time issue managing family, work, and everyday life at the same time. This OPR enables the combination of all this, and therefore it might recruit patients who otherwise would not have been able to or afford time to attend a rehabilitation program.

### Strengths and Limitations

The strengths of the study are the large sample size and the use of validated questionnaires. The major limitation is lack of a control group and therefore no causal conclusions can be drawn. We do not know if the improvements we observe could be explained from the intervention or which aspect of the multidisciplinary program provided the most benefits. Additional to the main components in the program (patient education, group conversation, and physical activity), the meeting of others in the same situation, group dynamic/unity, inspiration, and motivation could also play a role. Another limitation is that the majority of the patients in our sample had high education and lived as a couple, which might limit generalizability of our results to the broader breast cancer patient population. Future studies should include a control group that could confirm the outcome effects. In terms of more sustainable long-term effects, a follow-up systematic telephone motivational interview combined with a home-based physical activity program subsequently after the ORP would be of interest to investigate.

## Conclusion

In conclusion, HRQoL, fatigue, physical activity level, and physical capacity improved in women aged 30–65 years recently treated for BC who participated in a 1 day a week for 7 weeks of ORP. The short ORP used in this study may be cost- and time-effective both for patients and the society. Further studies with a control group are needed to confirm that a time- and cost-effective program for women with BC within working age could be beneficial.

## Supplementary Information

Below is the link to the electronic supplementary material.

Supplementary file1 (PDF 410 KB)

Supplementary file2 (PDF 21 KB)

Supplementary file3 (PDF 18 KB)

Supplementary file4 (PDF 21 KB)

Supplementary file5 (PDF 18 KB)

## Data Availability

The data that support the findings of this study are available on request from the corresponding author. The data are not publicly available due to privacy and ethical restrictions.
