# Supplementary material for: Health-Related Quality of Life, Fatigue, Level of Physical Activity, and Physical Capacity Before and After an Outpatient Rehabilitation Program for Women Within Working Age Treated for Breast Cancer
Source: J Cancer Educ. 2022 Aug 16;38(3):948–56. doi: 10.1007/s13187-022-02211-6 (PMC10234893; doi:10.1007/s13187-022-02211-6)
Supplement: Supplementary file 1 — Supplementary file1 (PDF 410 KB) [file 13187_2022_2211_MOESM1_ESM.pdf]

Online Resource 1

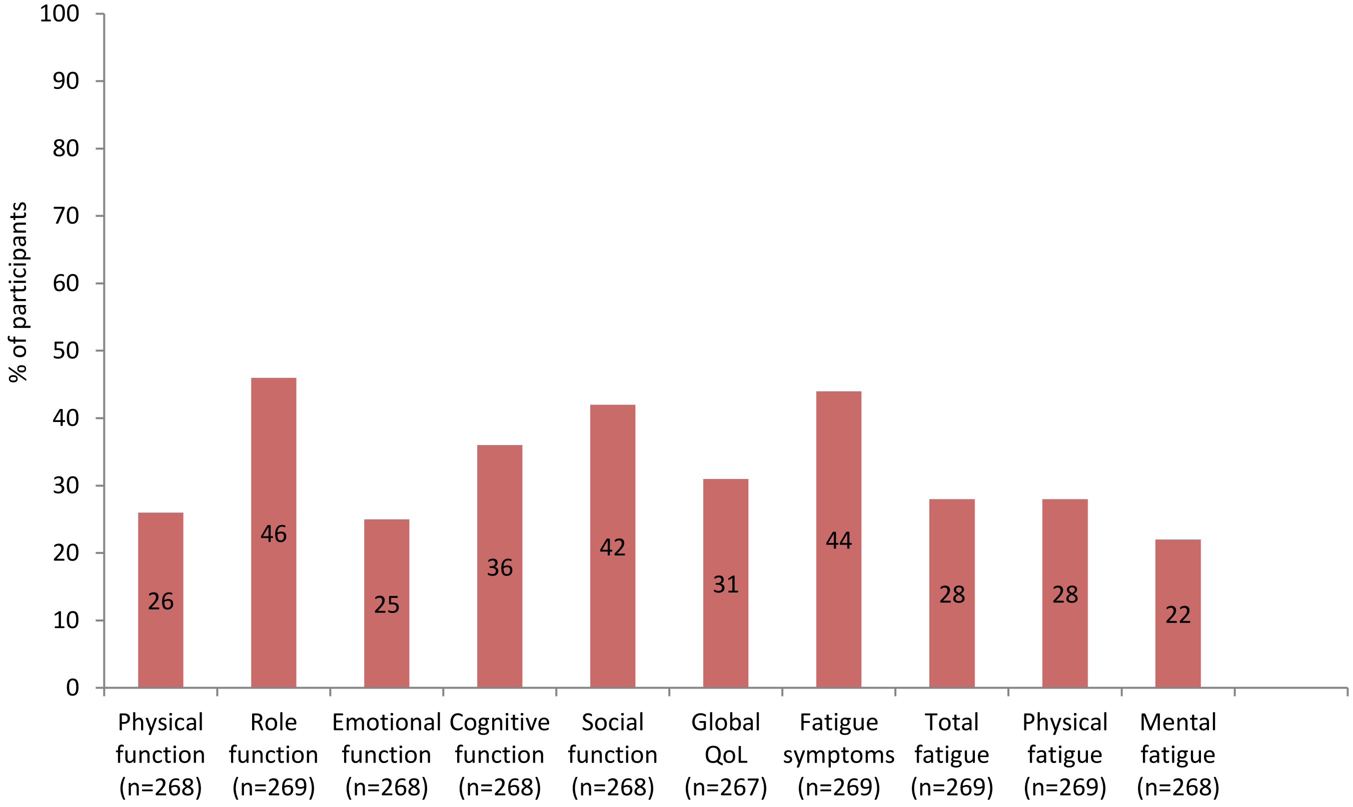

Supplementary Fig. 1 Proportion of participants with a clinical improvement during the outpatient rehabilitation program in health related quality of life (HRQoL) and fatigue

Clinical improvement: $\geq$ 10 points on HRQoL scales, $\geq$ 3.3 point on total fatigue, $\geq$ 2.1 point on physical fatigue and $\geq$ 1.2 point on mental fatigue.

HRQoL assessed by EORTC QLQ-C30: European Organization for Research and Treatment of Cancer Quality of Life Questionnaire-C30 and fatigue assessed by Fatigue Quesionnaire.
