# Supplementary material for: Health-Related Quality of Life, Fatigue, Level of Physical Activity, and Physical Capacity Before and After an Outpatient Rehabilitation Program for Women Within Working Age Treated for Breast Cancer
Source: J Cancer Educ. 2022 Aug 16;38(3):948–56. doi: 10.1007/s13187-022-02211-6 (PMC10234893; doi:10.1007/s13187-022-02211-6)
Supplement: Supplementary file 2 — Supplementary file2 (PDF 21 KB) [file 13187_2022_2211_MOESM2_ESM.pdf]

Online Resource 2

Supplementary Table 1. Factors associated with clinical improvement (versus no clinical improvement) in physical function

	Clinical improvement in physical function		Unadjusted			Adjusted[b]		
**Variables**	**Yes**	**No**	**cOR**	**95% CI**	***p***	**aOR**	**95% CI**	***p***
n (%)	70 (26)	198 (74)						
Baseline score physical function[a], mean (SD)	62.1 (14.7)	83.0 (12.9)	0.91	0.89-0.93	**<0.001**	0.91	0.87-0.94	**<0.001**
Age, mean (SD)	50.7 (8.1)	50.3 (7.0)	1.009	0.97-1.05	0.637			
Civil status, n (%)								
Living as a couple	48 (24)	152 (76)	1.0					
Living alone	22 (32)	46 (68)	1.51	0.83-2.77	0.177			
Education, n (%)								
High (> 13 years)	48 (24)	151 (76)	1.0					
Low (≤ 13 years)	21 (31)	46 (69)	1.44	0.78-2.64	0.245			
Months since diagnosis, mean (SD)	10.6 (2.3)	10.6 (2.7)	1.004	0.91-1.11	0.245			
Months since radiotherapy, mean (SD)	2.4 (1.6)	2.2 (1.5)	1.08	0.90-1.29	0.407			
Treatment, n (%)								
Non-systemic	1 (8)	11 (92)	1.0					
Systemic	69 (27)	187 (73)	4.06	0.51-32.03	0.18			
Comorbidity								
No	45 (21)	165 (79)	1.0			1.0		
Yes	25 (44)	32 (56)	2.87	1.54-5.32	**0.001**	1.41	0.63-3.14	0.401
Smoking (daily or occupationally)								
No	64 (26)	183 (74)	1.0					
Yes	6 (29)	15 (71)	1.14	0.43-3.07	0.790			
Overweight/obese (BMI (kg/m^2)≥25)								
No	27 (18)	122 (82)	1.0			1.0		
Yes	37 (36)	67 (64)	2.5	1.40-4.45	**0.002**	1.33	0.65-2.7	0.438

OR crude odds ratio; aOR adjusted odds ratio. 95% CI, 95% Confidence Interval.

[a]Increasing scores imply better function.

[b]Numbers included in the multivariate analyses were 252.
