# Supplementary material for: Health-Related Quality of Life, Fatigue, Level of Physical Activity, and Physical Capacity Before and After an Outpatient Rehabilitation Program for Women Within Working Age Treated for Breast Cancer
Source: J Cancer Educ. 2022 Aug 16;38(3):948–56. doi: 10.1007/s13187-022-02211-6 (PMC10234893; doi:10.1007/s13187-022-02211-6)
Supplement: Supplementary file 3 — Supplementary file3 (PDF 18 KB) [file 13187_2022_2211_MOESM3_ESM.pdf]

Online Resource 3

Supplementary Table 2. Factors associated with clinical improvement (versus no clinical improvement) in emotional function

	Clinical improvement in emotional function		Unadjusted			Adjusted[b]		
**Variables**	**Yes**	**No**	**cOR**	**95% CI**	***p***	**aOR**	**95% CI**	***p***
n (%)	67 (25)	201 (75)						
Baseline score emotional function[a], mean (SD)	51.9 (21.6)	78.2 (20.9)	0.95	0.94-0.97	**<0.001**	0.95	(0.94-0.97)	**<0.001**
Age, mean (SD)	50.5 (7.5)	50.3 (7.3)	1.003	0.97-1.04	0.893			
Civil status, n (%)								
Living as a couple	44 (22)	157 (78)	1.0			1.0		
Living alone	23 (34)	44 (66)	1.87	1.02-3.42	**0.043**	1.52	0.74-3.13	0.255
Education, n (%)								
High (> 13 years)	42 (21)	157 (79)	1.0			1.0		
Low ($\leq$ 13 years)	24 (36)	43 (64)	2.09	1.14-3.82	**0.017**	2.76	1.34-5.68	**0.006**
Months since diagnosis, mean (SD)	10.8 (2.3)	10.6 (2.7)	1.03	0.92-1.14	0.634			
Months since radiotherapy, mean (SD)	2.5 (1.4)	2.2 (1.6)	1.14	0.96-1.36	0.135			
Treatment, n (%)								
Non-systemic	3 (25)	9 (75)	1.0					
Systemic	64 (25)	192 (75)	1.0	0.26-3.81	1.0			
Comorbidity								
No	52 (25)	157 (75)	1.0					
Yes	15 (26)	43 (74)	1.05	0.54-2.05	0.879			
Smoking (daily or occupationally)								
No	60 (24)	187 (76)	1.0					
Yes	7 (33)	14 (67)	1.56	0.60-4.04	0.361			
Overweight/obese (BMI (kg/m^2)$\geq$25)								
No	34 (23)	116 (77)	1.0					
Yes	29 (28)	74 (72)	1.34	0.75-2.34	0.322			

OR crude odds ratio; aOR adjusted odds ratio. 95% CI, 95% Confidence Interval.

[a]Increasing scores imply better function.

[b]Numbers included in the multivariate analyses were 266.
