# Supplementary material for: Health-Related Quality of Life, Fatigue, Level of Physical Activity, and Physical Capacity Before and After an Outpatient Rehabilitation Program for Women Within Working Age Treated for Breast Cancer
Source: J Cancer Educ. 2022 Aug 16;38(3):948–56. doi: 10.1007/s13187-022-02211-6 (PMC10234893; doi:10.1007/s13187-022-02211-6)
Supplement: Supplementary file 4 — Supplementary file4 (PDF 21 KB) [file 13187_2022_2211_MOESM4_ESM.pdf]

Online Resource 4

Supplementary Table 3. Factors associated with clinical improvement (versus no clinical improvement) in physical fatigue

Clinical improvement in physical fatigue			Unadjusted			Adjusted[b]		
**Variables**	**Yes**	**No**	**cOR**	**95% CI**	***p***	**aOR**	**95% CI**	***p***
n (%)	76 (28)	193 (72)						
Baseline score physical fatigue[a], mean (SD)	15.6 (3.4)	12.9 (3.4)	1.27	1.16-1.39	**<0.001**	1.25	1.15-1.37	**<0.001**
Age, mean (SD)	50.7 (7.5)	50.2 (7.3)	1.009	0.97-1.05	0.618			
Civil status, n (%)								
Living as a couple	59 (29)	142 (71)	1.0					
Living alone	17 (25)	51 (75)	0.8	0.43-1.5	0.491			
Education, n (%)								
High (> 13 years)	51 (25)	149 (75)	1.0					
Low (≤ 13 years)	24 (36)	43 (64)	1.63	0.90-2.95	0.105			
Months since diagnosis, mean (SD)	10.4 (2.3)	10.7 (2.7)	0.96	0.87-1.07	0.481			
Months since radiotherapy, mean (SD)	2.3 (1.4)	2.2 (1.6)	1.04	0.87-1.25	0.634			
Treatment, n (%)								
Non-systemic	4 (33)	8 (67)	1.0					
Systemic	72 (28)	185 (72)	0.78	0.23-2.67	0.69			
Comorbidity								
No	58 (27)	153 (73)	1.0					
Yes	18 (32)	39 (68)	1.22	0.65-2.3	0.544			
Smoking (daily or occupationally)								
No	72 (29)	176 (71)	1.0					
Yes	4 (19)	17 (81)	0.58	0.19-1.77	0.334			
Overweight/obese (BMI (kg/m^2)≥25)								
No	35 (24)	114 (76)	1.0			1.0		
Yes	38 (36)	67 (64)	1.85	1.07-3.2	**0.029**	1.57	0.88-2.82	0.130

OR crude odds ratio; aOR adjusted odds ratio. 95% CI, 95% Confidence Interval.
[a]Increasing scores imply more fatigue.
[b]Numbers included in the multivariate analyses were 254.
