# Supplementary material for: Health-Related Quality of Life, Fatigue, Level of Physical Activity, and Physical Capacity Before and After an Outpatient Rehabilitation Program for Women Within Working Age Treated for Breast Cancer
Source: J Cancer Educ. 2022 Aug 16;38(3):948–56. doi: 10.1007/s13187-022-02211-6 (PMC10234893; doi:10.1007/s13187-022-02211-6)
Supplement: Supplementary file 5 — Supplementary file5 (PDF 18 KB) [file 13187_2022_2211_MOESM5_ESM.pdf]

Online Resource 5

Supplementary Table 4. Factors associated with clinical improvement (versus no clinical improvement) in mental fatigue

	**Clinical improvement in mental fatigue**		**Unadjusted**			**Adjusted[b]**		
**Variables**	**Yes**	**No**	**cOR**	**95% CI**	***p***	**aOR**	**95% CI**	***p***
n (%)	59 (22)	209 (78)						
Baseline score mental fatigue[a], mean (SD)	8.5 (2.0)	6.8 (1.9)	1.57	1.34-1.85	**<0.001**	1.55	1.31-1.82	**<0.001**
Age, mean (SD)	50.2 (7.9)	50.4 (7.2)	0.99	0.96-1.04	0.820			
Civil status, n (%)								
Living as a couple	36 (18)	165 (82)	1.0			1.0		
Living alone	23 (34)	44 (66)	2.4	1.29-4.45	**0.006**	2.02	1.03-3.97	**0.040**
Education, n (%)								
High (> 13 years)	46 (23)	153 (77)	1.0					
Low (≤ 13 years)	13 (19)	54 (81)	0.8	1.4-1.6	0.527			
Months since diagnosis, mean (SD)	10.3 (2.1)	10.7 (2.7)	0.95	0.84-1.06	0.351			
Months since radiotherapy, mean (SD)	2.2 (1.4)	2.3 (1.6)	0.96	0.79-1.17	0.689			
Treatment, n (%)								
Non-systemic	2 (17)	10 (83)	1.0					
Systemic	57 (22)	199 (78)	1.43	0.31-6.72	0.65			
Comorbidity								
No	49 (23)	161 (77)	1.0					
Yes	10 (18)	47 (82)	0.7	0.33-1.49	0.352			
Smoking (daily or occupationally)								
No	54 (22)	193 (78)	1.0					
Yes	5 (24)	16 (76)	1.12	0.39-3.19	0.836			
Overweight/obese (BMI (kg/m^2)≥25)								
No	33 (22)	115 (78)	1.0					
Yes	22 (21)	83 (79)	0.92	0.50-1.70	0.798			

OR crude odds ratio; aOR adjusted odds ratio. 95% CI, 95% Confidence Interval.

[a]Increasing scores imply more fatigue.

[b]Numbers included in the multivariate analyses were 268.
